# Supplementary material for: Tead1a Initiates Transcriptional Priming Through the TEAD1a/YAP‐Notch1‐Spi1/Cebpα Axis to Promote Neutrophil Fate
Source: Adv Sci (Weinh). 2025 Aug 28;12(41):e05441. doi: 10.1002/advs.202505441 (PMC12591127; doi:10.1002/advs.202505441)
Supplement: Supplementary file 4 — Supplementary Dataset [file ADVS-12-e05441-s004.zip › 3-4.zip!/3-4/02-4.cluster/cluster9/Enrich/ego_all.pdf]

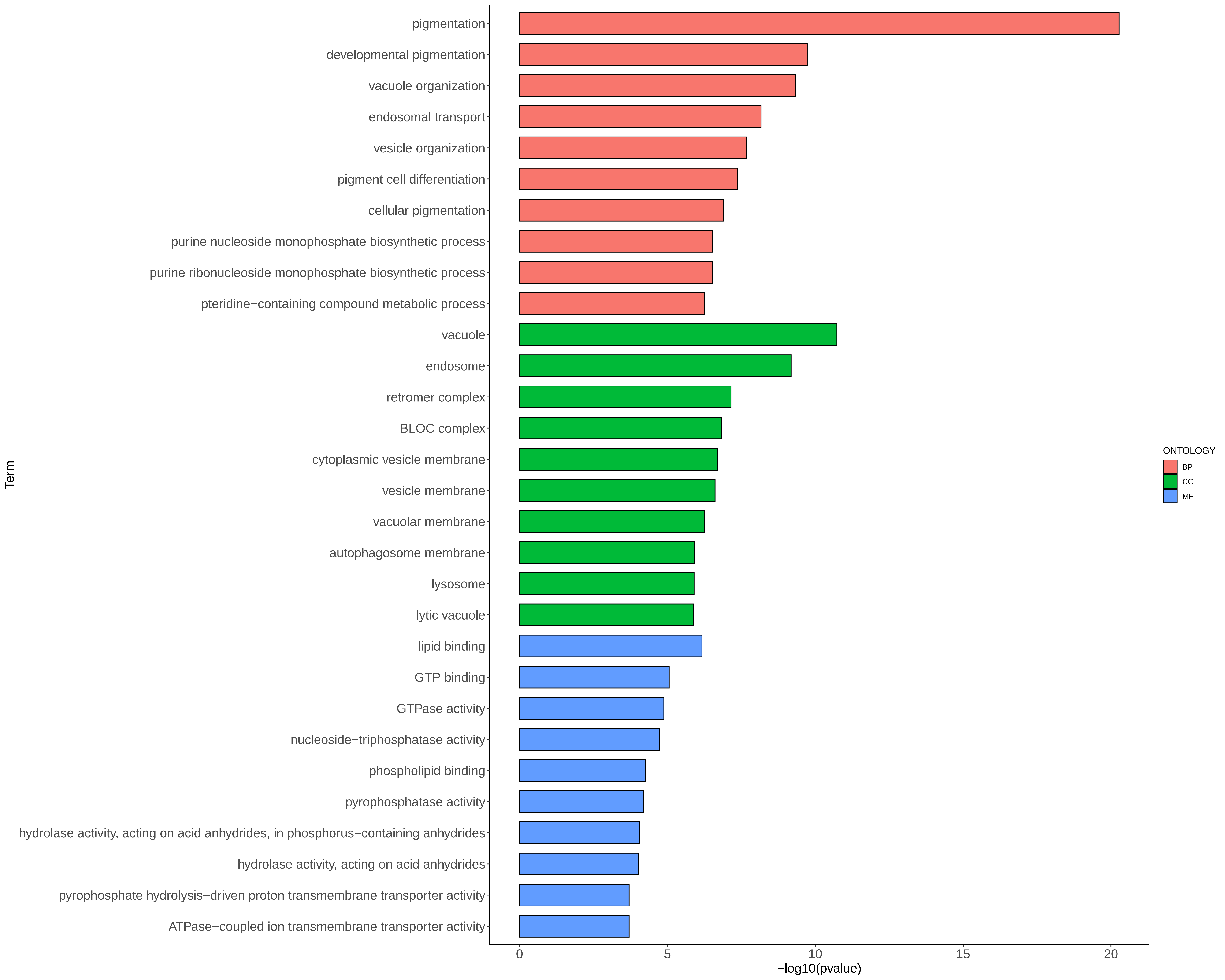

pigmentation
developmental pigmentation
vacuole organization
endosomal transport
vesicle organization
pigment cell differentiation
cellular pigmentation
purine nucleoside monophosphate biosynthetic process
purine ribonucleoside monophosphate biosynthetic process
pteridine−containing compound metabolic process
vacuole
endosome
retromer complex
BLOC complex
cytoplasmic vesicle membrane
vesicle membrane
vacuolar membrane
autophagosome membrane
lysosome
lytic vacuole
lipid binding
GTP binding
GTPase activity
nucleoside−triphosphatase activity
phospholipid binding
pyrophosphatase activity
hydrolase activity, acting on acid anhydrides, in phosphorus−containing anhydrides
hydrolase activity, acting on acid anhydrides
pyrophosphate hydrolysis−driven proton transmembrane transporter activity
ATPase−coupled ion transmembrane transporter activity
Term
0
5
10
15
20
−log10(pvalue)
ONTOLOGY
BP
CC
MF
